# Arthroscopically Assisted Combined Anterior and Posterior Cruciate Ligament Reconstruction with Autologous Hamstring Grafts–Isokinetic Assessment with Control Group

**DOI:** 10.1371/journal.pone.0082462

**Published:** 2013-12-30

**Authors:** Tomasz Piontek, Kinga Ciemniewska-Gorzela, Andrzej Szulc, Jakub Naczk, Martyna Wardak, Tadeusz Trzaska, Witold Dudzinski, Monika Grygorowicz

**Affiliations:** 1 Orthopaedic Department, Rehasport Clinic, Poznan, Poland; 2 Department of Pediatric Orthopaedics and Traumatology, Karol Marcinkowski University of Medical Sciences, Poznan, Poland; 3 Department of Traumatology of the Chair of Sports Medicine, University School of Physical Education in Poznan, Poland; 4 Research and Development Department, Rehasport Clinic, Poznan, Poland; Universidad Europea de Madrid, Spain

## Abstract

**Objective:**

The aim of the study was to: 1) evaluate the differences in pre-post operative knee functioning, mechanical stability, isokinetic knee muscle strength in simultaneous arthroscopic patients after having undergone an anterior cruciate ligament (ACL) and the posterior cruciate ligament (PCL) with hamstring tendons reconstruction, 2) compare the results of ACL/PCL patients with the control group.

**Design:**

Controlled Laboratory Study.

**Materials and Methods:**

Results of 11 ACL/PCL patients had been matched with 22 uninjured control participants (CP). Prior to surgery, and minimum 2 years after it, functional assessment (Lysholm and IKDC 2000), mechanical knee joint stability evaluation (Lachman and “drawer” test) and isokinetic tests (bilateral knee muscle examination) had been performed. Different rehabilitation exercises had been used: isometric, passive exercises, exercises increasing the range of motion and proprioception, strength exercises and specific functional exercises.

**Results:**

After arthroscopy no significant differences had been found between the injured and uninjured leg in all isokinetic parameters in ACL/PCL patients. However, ACL/PCL patients had still shown significantly lower values of strength in relative isokinetic knee flexors (p = 0.0065) and extensors (p = 0.0171) compared to the CP. There were no differences between groups regarding absolute isokinetic strength and flexors/extensors ratio. There was statistically significant progress in IKDC 2000 (p = 0.0044) and Lysholm (p = 0.0044) scales prior to (44 and 60 points respectively) and after the reconstruction (61 for IKDC 2000 and 94 points for Lysholm).

**Conclusions:**

Although harvesting tendons of semitendinosus and/or gracilis from the healthy extremity diminishes muscle strength of knee flexors in comparison to the CP, flexor strength had improved. Statistically significant improvement of the knee extensor function may indicate that the recreation of joint mechanical stability is required for restoring normal muscle strength. Without restoring normal muscle function and strength, surgical intervention alone may not be sufficient enough to ensure expected improvement of the articular function.

## Introduction

Damage to two or more ligaments within the knee joint leads to significant impairment of the articular function. The treatment of complex knee joint instability has attracted a lot of attention in recent years. There have been many research papers on the anatomy and biomechanics of knee ligaments, as well as mechanisms contributing to multiple ligament injuries, and secondary multidirectional instabilities of the knee joint [1], [2]. Numerous authors have described various, predominantly arthroscopic, techniques used in the combined multiligament surgical reconstruction of the knee joint when restoring its mechanical stability [1]–[17]; fewer of them have revealed the outcomes of such techniques being applied in surgical treatment of complex instability [7]–[9], [11], [13], [14], [17], and even fewer have conducted prospective studies with pre- and post-operative assessment [7]–[9] based on mechanical stability and knee joint subjective functional evaluation. There is still a lack of information about combining the evaluation of mechanical knee stability with subjective and objective functional joint assessment. Graft materials are another topic widely discussed in relevant publications [16]. The majority of authors refrain from using only hamstring autografts in the single-stage of ACL/PCL reconstruction, as they fear this might weaken knee flexors - and thus, delay rehabilitation [3], [5], [8], [11], [12]. Articles regarding the operative technique which was used in single-stage ACL/PCL reconstruction with hamstring autografts only, have reported no serious complications resulting from the graft being harvested from an uninjured limb [11], [12], [14], [17]. Other papers evaluating the strength of knee flexors in unaffected limbs, following tendon harvesting for ACL reconstruction in the contralateral knee, have shown certain flexor weakness, however, the differences are statistically insignificant [18]–[21]. There are no objective studies confirming the that graft harvesting from an unaffected limb does not impair muscle strength of relevant muscle groups, or lead to functional impairment of the limb [14]. Based on this knowledge, a hypothesis has been developed, stating that hamstring harvesting from both the injured and intact leg does not lead to flexor weakening. The second hypothesis of the present study is that good results could be achieved in the majority of patients by the implementation of our standardized surgical approach and postoperative protocol.

The aim of this study was to prospectively investigate the recovery of knee function and isokinetic strength after multiple-knee-ligament reconstruction with autologous hamstring grafts in patients after having undergone arthroscopic assisted combined ACL reconstruction of the anterior cruciate ligament (ACL), and posterior cruciate ligament (PCL) with authogenous hamstring tendons. Afterwards patients had been compared with their un-operated side, as also with healthy subjects.

## Materials and Methods

### Subjects

Between 2006 and 2008, all patients who had undergone multiple-knee-ligament reconstruction after a traumatic knee injury, had been considered for the inclusion in the study. Patients with ipsilateral lower-limb injuries, or those who had experienced a concurrent injury to the contralateral knee had been excluded. The present study presents the results of a prospective study with 11 patients having undergone surgical treatment due to anteroposterior instability of the knee (ACL/PCL). Within the period of the study, 12 patients had been treated arthroscopically, using combined anterior and posterior cruciate ligament reconstructions. Eleven of these cases have been discussed in the presented paper. One case had been excluded from the study, due to an injury 1 year after the multiligament reconstruction. The study had included 8 male and 3 female patients (Tab. 1). In nine subjects ligament damage had been found in the right lower extremity. The remaining 2 cases were of the left knee. All subjects had chronic damage to both cruciate ligaments of the knee (6 and more months from injury to surgery). Four patients had had additional tibial collateral ligament reconstruction. In two cases the medial meniscus had been sutured. In one case, a patient had had lateral partial menisectomy. Four cases of ligament injury had been caused by traffic accidents, two others by a fall from high altitude, and the remaining five patients had experienced ligament damage as a result of sports injuries. In all treated patients ligament reconstruction had been performed by means of hamstring autografts harvested from the injured extremity (in all patients ST and GR tendons), and from the unaffected limb (in 4 patients ST tendons was only harvested, in 7 cases – both ST and GR tendons). If the tendon diameter had been larger than 7mm, we would have harvested only ST; otherwise, we had decided to harvest both ST and GR tendons.

**Table 1:** Descriptive statistics of patients and control subjects participating in the study.

Feature	Group	Median ± SEM	p value of Mann Whitney U Test
Age [yrs]	ACL/PCL patients (n = 11)	36.00±3.82	p = 0.2169
	Control (n = 11)	26.00±2.79	
Weight [kg]	ACL/PCL patients (n = 11)	78.00±3.33	p = 0.9487
	Control (n = 11)	76.00±4.56	
Height [cm]	ACL/PCL patients (n = 11)	171.00±2.43	p = 0.2169
	Control (n = 11)	171.00±3.46	
BMI	ACL/PCL patients (n = 11)	25.30±0.97	p = 0.0651
	Control (n = 11)	22.20±1.05	

Patients' results had been compared with the control group of 11 healthy volunteers (7 males and 4 females). Descriptive statistics of all participants have been presented in table 1.

### Clinical evaluation

Pre- and postoperative evaluation of subjective knee symptoms had been conducted by median of the Lysholm knee score and IKDC 2000. This was followed by bilateral objective functional assessment of extensors and flexors using the isokinetic test on the Biodex 3 dynamometer (System 3: Biodex Medical System, Shirley, NY). A total translation of the tibia, relative to the femur with the knee in a 30° flexion (“Lachman test”) and in a 90° flexion (“drawer” test), had been measured. Knee laxity we assessed before and after ACL/PCL reconstructive surgery using arthrometer “Rolimeter” (Aircast). Investigations were conducted by independent specialists.

### Surgical technique

All arthroscopic reconstructions of combined ACL/PCL rupture had been performed by the same surgeon at our institution with autogenous hamstring tendons in 1 stage in a 4-tunnel manner. The semitendinosus tendon and gracilis tendon from the uninjured leg was used to make two 4-stranded grafts to reconstruct the PCL, and those from the injured leg were used to make two 4-stranded grafts to reconstruct the ACL. The surgical technique has previously been described in detail, in Polish, by Piontek et. all. [15]. The patient is positioned supine on the operating table with a well-padded tourniquet placed over the both thighs. Anterolateral and anteromedial arthroscopic portals are established. The ACL, PCL, and menisci are visualized to confirm the physical examination findings. Once this is done, the ACL is debrided, the PCL stay intact.

**Figure 1:**
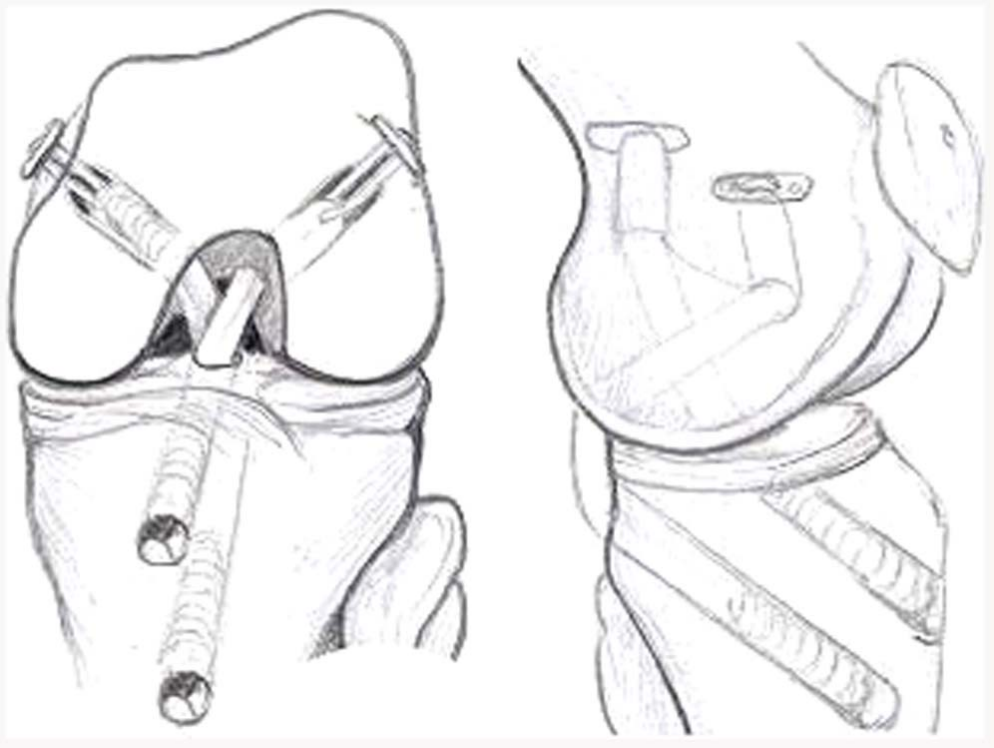
Combined ACL/PCL reconstruction using autogenous ST GR grafts. The grafts were placed through the bony tunnels, which were precisely created to reproduce the anatomic insertion site of the bundle of the PCL, and the anatomic insertion site of the ACL ,which were simultaneously secured with the endo-button technique in femur and BioRCI screw in the tibia. Additionally, for better fixation of the PCL graft, the BioRCI screw was placed in the femur tunnel.

**Figure 2:**
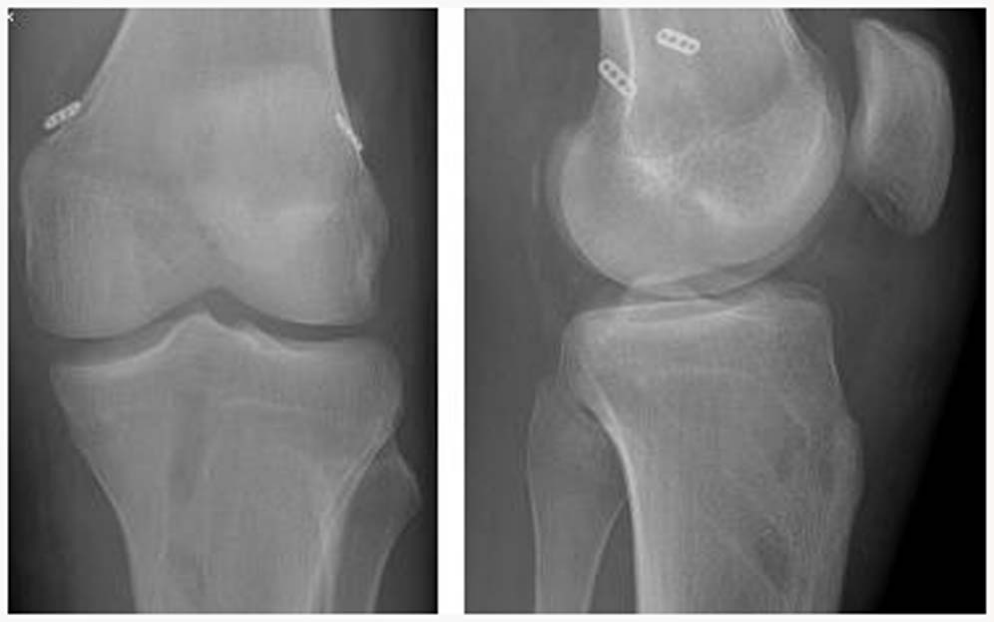
Radiographs after simultaneous ACL and PCL reconstruction.

The hamstring tendons of the injured leg are palpated to insure a good position of the anteromedial, vertical incision. The incision is made and the soft tissue is dissected to the level of the Sartorius fascia. The semitendinosus and gracilis is harvested. This same incision will serve as tibial tunnel entry sites for the ACL and PCL grafts. The hamstring tendons of the uninjured leg are palpated to insure a good position of the anteromedial, horizontal incision. The incision is made and the semitendinosus and gracilis is harvested. The incision is closed with a drainage. The PCL tibial and femoral tunnels are created with the help of a PCL/ACL drill guide (Smith&Nephew, Andover, USA). The transtibial PCL tunnel goes from the anteromedial aspect of the proximal tibia, 1 cm below the tibial tubercle to the exit in the inferior lateral aspect of the PCL anatomic insertion site. The PCL femoral tunnel originates externally between the medial femoral epicondyle and the medial femoral condylar articular surface to emerge through the centre of the stump of the anterolateral bundle of the posterior cruciate ligament. The ACL tibial tunnel begins externally at a point being 1 cm proximal to the tibial tubercle on the anteromedial surface of the proximal tibia to emerge through the centre of the stump of the ACL tibial footprint. The femoral tunnel is positioned next to the over-the-top position on the medial wall of the lateral femoral condoyle, on the ACL anatomic insertion site. The tunnel is created to leave a 1 to 2 mm posterior cortical wall so that interference fixation can be used. The PCL and ACL grafts are positioned and anchored on the femoral side, using the endobutton technique, and left free on the tibial side. The PCL graft is additionally anchored on the femoral side with the interference screw. Attention is then turned to the collateral ligaments. The lateral or medial horizontal incision is made depending on the needs. MCL or PLC is reconstructed with a free authologus gracilis graft. The knee is then placed in a 30° of flexion and a collateral MCL or PLC graft is fixed with interference screws. The knee is placed in 70° to 90° flexion. A firm anterior drawer force is applied to the proximal tibia to restore the normal tibial step-off, and fixation is achieved on the tibial side of the PCL graft with a interference screw. Finally, the knee is placed in 15–30° flexion, with tension on the ACL graft, and final fixation is achieved of the ACL graft with a interference screw. Figure 1 and 2 present the placement of bone tunnels and the type of graft fixation.

### Postoperative management

The operated extremity had been placed in an orthosis with posterior tibia support, stabilizing the knee in a 30° flexion. Postoperative hospitalization had lasted no longer than two days. Management of pain and swelling in the initial hours after surgery had been executed by means of cold packs, limb elevation and administration of analgesics. On the first day after surgery, patients initiated proprioception exercises, with isometric exercises of the quadriceps being introduced on the second day after surgical intervention. Starting from the second day, post-surgery patients could walk with elbow crutches and without weight bearing on the operated limb. The knee had remained immobilized in the postoperative brace (positioned as above) for a period of 2 weeks. Exercises increasing the range of motion (ROM) were introduced next, so full extension and a 90° flexion was possible to be achieved within the following 3 weeks. From the 6th week, muscle strength exercises for knee joint flexors had been added. A full ROM had been obtained within the 8–10 week. Walking with full weight bearing with the support of postoperative knee orthosis had been initiated 6 weeks after the operation; 2 weeks later patients were able to manage without orthosis, and after the period of 4 months they were allowed to take up running. Orthopedic orthosis with posterior tibia support was applied at bedtime within the period of 3 months after the operation. Patients had returned to full athletic activity and heavier work after having regained sufficient muscle strength and range of motion, and had achieved good functional test results. This was typically observed after an average 8 months of rehabilitation.

### Statistics

The characteristics of the studied variables have been presented with their respective measures of descriptive statistics. The authors have conducted a statistical analysis of objective (absolute and relative isokinetic knee flexors and extensor strength, mechanical knee stability - Lachman and “drawer” tests with arthrometer measurements) and subjective (IKDC 2000 and Lysholm knee scoring scales) variables usually used in the assessment of ACL reconstructed patients. Normality of distribution had been verified using the Shapiro-Wilk test. There was no normal distribution, and hence quantitative variables had been presented using the median ± standard error (SEM) and non-paramteric tests had been used in further analysis. The Wilcoxon signed rank test had been used to determine the significant differences between objective and subjective parameters prior to reconstruction, and in follow-up examinations. To compare isokinetic and mechanical parameters of the injured limb with the non-injured limb, and with the control group, the Mann-Whitney U test had been calculated. The correlation between specific results (isokinetic parameters, subjective scores and joint stability outcomes) had been determined using the Spearman's rank correlation test. Statistical significance was set at p<0.05. Statistical analysis had been performed using Statistica v. 7.1 software.

### Ethics

The study was performed with the approval of the local research ethics committee (Bioethics Committee at the Karol Marcinkowski Poznan University of Medical Sciences), in accordance with the Declaration of Helsinki, and all participants had provided their written informed consent of participation in this study.

## Results

**Figure 3:**
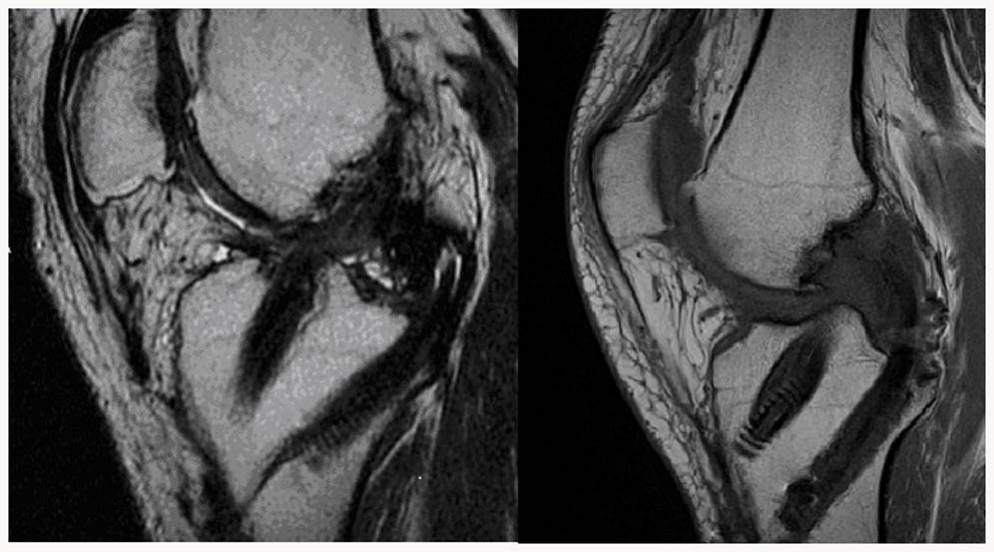
MRI of a knee 5 years after a simultaneous arthroscopic reconstruction of the ACL and the PCL with hamstring tendons showing an proper localization and structure of grafts without knee subluxation.

In the follow up examination, the proper localization and structure of grafts had been found on MRI scans in all patients (Figure 3). The results of the clinical examination and subjective assessment have been presented in tables 2–5. The average follow-up period amounted to 27 months±4.04 SEM.

**Table 2:** Absolute and relative isokinetic strength in ACL/PCL patients.

Isokinetic parameter	Tested limb	PRE - operatively		Follow - up	
		Median ± SEM	p value of Mann Whitney U Test	Median ± SEM	p value of Mann Whitney U Test
Absolute extensors strength	injured (n = 11)	106.00±16.53	**p = 0.0158**	175.00±18.39**	p = 0.3653
	uninjured (n = 11)	157.00±13.96		164.50±18.27	
Absolute flexors strength	injured (n = 11)	62.00±17.41	p = 0.0759	91.20±9.40	p = 0.6063
	uninjured (n = 11)	86.00±7.45		96.00±10.20	
Relative extensors strength	injured (n = 11)	1.32±0.21	**p = 0.0233**	1.91±0.26**	p = 0.1932
	uninjured (n = 11)	1.91±0.17		2.24±0.21	
Relative flexors strength	injured (n = 11)	0.83±0.19	p = 0.0652	1.18±0.11	p = 0.5190
	uninjured (n = 11)	1.10±0.07		1.20±0.13	
Flexors/Extensors ratio	injured (n = 11)	0.63±0.07	p = 0.2426	0.55±0.03	p = 0.2426
	uninjured (n = 11)	0.54±0.02		0.52±0.02	

significant time effects. Wilcoxon signed rank test; (p = 0.0033).

**Table 3:** Absolute and relative isokinetic strength in control group.

Isokinetic parameter	Tested limb	Control group	
		Median ± SEM	p value of Mann Whitney U Test
Absolute extensors strength	dominant (n = 11)	206.50±21.34	p = 0.9487
	non-dominant (n = 11)	183.70±18.37	
Absolute flexors strength	dominant (n = 11)	124.40±12.14	p = 0.7477
	non-dominant (n = 11)	118.80±11.47	
Relative extensors strength	dominant (n = 11)	2.81±0.17	p = 0.8977
	non-dominant (n = 11)	2.76±0.17	
Relative flexors strength	dominant (n = 11)	1.60±0.11	p = 0.8470
	non-dominant (n = 11)	1.49±0.10	
Flexors/Extensors ratio	dominant (n = 11)	0.53±0.02	p = 0.6994
	non-dominant (n = 11)	0.51±0.04	

**Table 4:** Absolute and relative isokinetic strength in ACL/PCL patients vs. control group.

Isokinetic parameter	Group	Tested limb	Median ± SEM	p value of Mann Whitney U Test
Absolute extensors strength	ACL/PCL group	injured (n = 11)	175.00±18.39	p = 0.2042
	control group	both (n = 22)	184.95±13.75	
Absolute flexors strength	ACL/PCL group	injured (n = 11)	91.20±9.40	p = 0.1658
	control group	both (n = 22)	120.35±8.16	
Relative extensors strength	ACL/PCL group	injured (n = 11)	1.91±0.26	**p = 0.0171**
	control group	both (n = 22)	2.79±0.12	
Relative flexors strength	ACL/PCL group	injured (n = 11)	1.18±0.11	**p = 0.0065**
	control group	both (n = 22)	1.52±0.07	
Flexors/Extensors ratio	ACL/PCL group	injured (n = 11)	0.55±0.03	p = 0.9252
	control group	both (n = 22)	0.52±0.02	

**Table 5:** Mechanical knee stability in ACL/PCL patients.

Mechanical stability	Tested limb	PRE - operatively		Follow - up	
		Median ± SEM	p value of Mann Whitney U Test	Median ± SEM	p value of Mann Whitney U Test
Lachman	injured (n = 11)	16.00±1.01	**p<0.0001**	7.00±0.45**	p = 0.0652
	uninjured (n = 11)	6.00±0.84		6.00±0.43	
“drawer test”	injured (n = 11)	18.00±0.90	**p<0.0001**	6.00±0.73**	p = 0.0759
	uninjured (n = 11)	4.00±0.52		5.00±0.34	

significant time effects. Wilcoxon signed rank test; (p = 0.0033).

### Objective knee evaluation

Prior to the reconstruction, statistically significant differences between the injured and non-injured leg in knee extensor isokinetic strength had been found. The values of the extensor peak torque (absolute isokinetic strength) and extensor peak torque/body weight (relative isokinetic strength) had been significantly lower in the injured knee. In the follow up assessment no significant differences between both legs in all isokinetic parameters in ACL/ACL patients had been achieved (Table 2). Due to the fact, that no significant differences between the dominant and non-dominant limb in the control group had been noted (table 3), we decided to compare the isokinetic results achieved by the reconstructed patients, with the mean value of isokinetic extensor strength from both legs in the control group (table 4). After the arthroscopy and ACL/PCL rehabilitation program, patients had still significantly lower values in relative isokinetic knee flexors (p = 0.0065) and extensors (p = 0.0171) in comparison to the control group. However, there were no differences between the groups regarding absolute isokinetic strength and knee flexor/extensor ratio. Assessment prior to the reconstruction had revealed that the total translation of the tibia, relative to the femur, had been significantly different in the limb with a cruciate ligament injury, than in the unaffected limb. Statistically higher values for the Lachman (p<0.0001) and “drawer” test (p<0.0001) in the injured compared to the non-injured limb in ACL/PCL patients had been noted (table 5). In the follow up evaluation there were still differences in the reconstructed and non-reconstructed limb in both these tests. However, they were not significantly important. The Wilcoxon signed rank test indicates that there was a significant time effect in the absolute (p = 0.0033) and relative (p = 0.0033) isokinetic knee extensor strength - Lachman (p = 0.0033) and “drawer” test (p = 0.0033). For flexor strength and knee flexion/extension ratio, there were no statistically significant differences between pre- and post-reconstruction.

### Subjective knee symptoms

**Figure 4:**
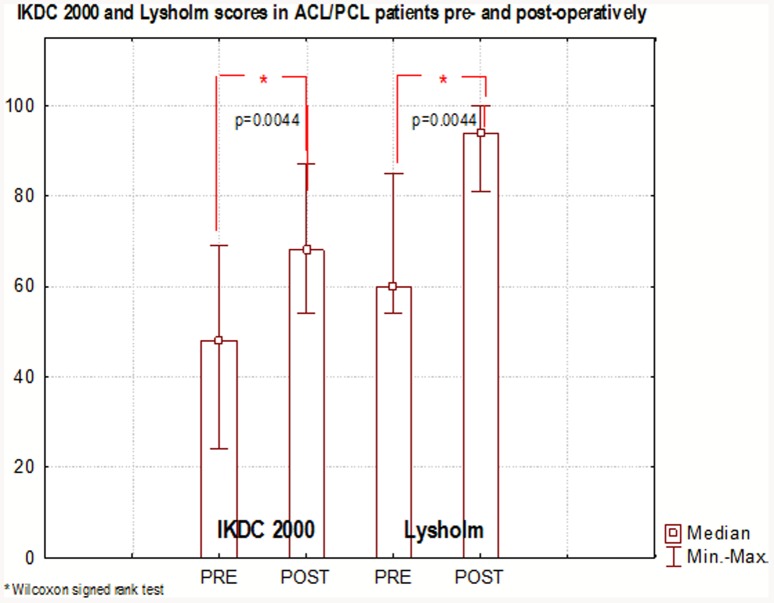
Knee function subjective analysis in ACL/PCL patients.

Time effects for subjective knee scoring scales in ACL/PCL patients had also been tested. The Wilcoxon signed rank test had confirmed that there was statistically significant progress in IKDC 2000 (p = 0.0044) and Lysholm (p = 0.0044) scales (figure 4) prior to (44 and 60 points respectively) and after the reconstruction (61 for IKDC 2000 and 94 points for Lysholm). According to the subjective Lysholm knee score 6 subjects (54,60%) had obtained excellent results (between 98–100 points) and 5 subjects (45,40%) had reported very good results (between 93–97 points). There were no acceptable or poor scores. In the IKDC 2000 scoring system, four excellent results (36,40%) had been reported, with six very good (54,60%) and one acceptable score (9%). During the post-surgical tests a few complications had also been noted: one patient (9%) had developed arthrofibrosis, requiring surgical removal one year after the reconstruction. All treated subjects had experienced skin sensation disturbances (100%) in the shins of both legs, in the proximity of scarring at the graft harvesting site. Normal sensation had completely been restored in the period of 1 year after surgery among eight patients (72,80%). Disturbances of sensation persisted in the form of minor hyperesthesia, and temporary paresthesia, among the remaining three patients (27,20%).

### Correlations between test results

Patients with more favourable results of isokinetic extensor muscle assessment prior to treatment, had achieved better results in the follow-up examination (r = 0,6 p = 0,03). In follow-ups, results of isokinetic extensor muscle assessment are correlated with subjective scores (r = 0,6 p = 0,04). The better the parameters of muscle work, the more favourable the functional result is. The results of isokinetic muscle assessment are not correlated with the results of mechanical stabilizing in both tests (Lachman and “drawer test”). The results of subjective score assessment are also not correlated with the results of mechanical stabilizing.

## Discussion

The main finding of the current paper is that although the majority of tested parameters of the reconstructed leg had improved - the relative isokinetic knee extensors and flexor strength deficits persist (in the follow-up assessment) in patients with combined ACL/PCL chronic ligament lesion in comparison to the control group. Pre-post reconstruction improvement of absolute and relative knee flexor strength of the non-reconstructed leg after hamstring harvesting had been observed. However, the deficit persists in comparison to the control group (for the relative parameter). The question arises as to whether or not it is safe to harvest the hamstring from the healthy leg. Ibrahim et al. and Zhao et al. have analyzed data from patients subjected to combined arthroscopic reconstruction of ACL/PCL with autologous hamstring grafts harvested from the healthy and the injured limb. Only one case of hematoma in the area of graft harvesting [11] and minor pain in one patient [17] had been reported. In our analysis 8 cases had revealed no complications associated with the graft harvesting site.

Isokinetic muscle strength had been restored almost completely, however a relative strength deficit had remained. In the material collected by Kartus et al., regeneration of semitendinosus and gracilis tendons had been observed 2 years after graft collection [22]. Similar findings had been reported by Nakamura et al. [23]. In Jenkins et all. the study group had recovered to 85% of the uninjured side within two years, and hamstrings had recovered to 90%. Hamstring recovery was faster than quadriceps, but at final recovery there were no overall difference between the muscle group recovery. They had revealed that deficits persist in comparison with the uninjured limb [24]. The above considerations suggest that applying only autologous hamstring grafts in combined arthroscopic ACL and PCL reconstruction is associated with a few complications. Furthermore, the surgical technique required for this procedure is simple, thus results in making this type of graft more useful in relation to other materials. Our materials have revealed that among patients with a history of ACL and PCL reconstruction, the results of subjective scores had been correlated with extensor muscle strength results. Patients achieving better results in the isokinetic muscle function evaluation, had also been superior in subjective assessment. At the same time, better outcomes of isokinetic function evaluation prior to treatment were also helpful in achieving more favourable results in follow-up examinations. Perhaps specialized rehabilitation should be introduced preoperatively, in order to obtain optimized muscle strength and an appropriate postural control strategy for better outcomes of the surgical intervention.

Statistically significant improvement of the results of isokinetic assessment had been obtained among all patients, in all tests. As promising as this might seem, better results alone do not mean that such improvement is significant for the patient, and enables full functional recovery of the joint. Ibrahim et al. had encountered inconsistency in subjective assessment results in the Lysholm score and IKDC [11]. The main score in the Lysholm evaluation had proven excellent and good results, while in the IKDC assessment there were no excellent results, 10 good, 10 acceptable and 2 poor. This substantial irregularity of subjective scoring results further justifies the need to apply objective tests for the evaluation of the knee function. Fanelli et al. had conducted subjective evaluation of knee symptoms using three scoring tools prior to combined ACL and PCL reconstruction, and after follow-ups of over 2 years [8]. Statistically significant improvement had been noted from surgical intervention. Postoperative Lysholm, Tegner, and Hospital for Special Surgery knee ligament rating scale main values were 91.2, 5.3, and 86.8, respectively. This is comparable to the results obtained by other authors, as well as the outcomes of the present study.

In the study by Strobel et al. the main postoperative total anterior-posterior side-to-side difference with the KT-1000 arthrometer testing was 2.00±2.23 mm (range, −4 to 7 mm) [14]. Findings by Fanelli et al. had been equally favourable [8]. According to the data provided by the Fanelli team, the main difference of tibia translation. relative to the femur, was 2.7 mm. Comparable results, by means of 2.55 mm had been obtained in our subjects after follow-ups. Even though mechanical stability resulting from the ACL/PCL reconstruction was good, similarly to other authors we had failed to obtain grade A results in the IKDC assessment [7]–[9], [11], [13], [14], [17].

In order to perform a real evaluation of treatment outcomes in complex knee instability, it is necessary to compare the results with the control group. The strengths of this study were its prospective design, incorporating objective measures of muscle recovery recorded by an independent research physiotherapist, by using the patient's own uninjured leg as a paired control group. The proposed surgical technique for combined arthroscopic reconstruction of the anterior and posterior cruciate ligament using only hamstring autografts, ensures favourable mechanical stability of the knee joint. Both factors – mechanical stability and normal muscle function – are interconnected and influence the knee joint performance. Mechanical instability seems to change muscle tone control and lead to diminishing muscle strength of knee extensors.

## Study Limitations

The major limitation of this study is its small sample size. It has to be remembered that statistically significant findings from studies with small sample sizes should be treated with increased scepticism. However, there is nothing wrong with conducting well-designed small studies (especially to avoid using too many resources, e.g. subjects, time and financial costs, on finding an association between analyzed factors), they only need to be interpreted very carefully. Due to the small study group this study was also unable to examine the strength, movement and functional outcomes of various subgroups, such as acute versus delayed repair, or two- versus three-ligament reconstruction.

## Conclusions

Although harvesting tendons of semitendinosus and/or gracilis from the healthy extremity diminishes muscle strength of knee flexors in comparison to the healthy population, flexor strength had improved. Statistically significant improvement of the knee extensor function may indicate that the recreation of joint mechanical stability is required for restoring normal muscle strength. At the same time, considerable correlation of functional evaluation results, with the results of isokinetic muscle assessment, might suggest that without restoring normal muscle function and strength, surgical intervention alone may not be sufficient enough to ensure expected improvement of articular function.
